# Genetic and morphological divergence among three closely related *Phrynocephalus* species (Agamidae)

**DOI:** 10.1186/s12862-019-1443-y

**Published:** 2019-06-06

**Authors:** Chao-Chao Hu, Yan-Qing Wu, Li Ma, Yi-Jing Chen, Xiang Ji

**Affiliations:** 1 Jiangsu Key Laboratory for Biodiversity and Biotechnology, College of Life Sciences, Nanjing Normal University, Nanjing, 210023 Jiangsu China; 2 Analysis and Testing Center, Nanjing Normal University, Nanjing, 210023 Jiangsu China; 3 Nanjing Institute of Environmental Sciences, Ministry of Ecology and Environment, Nanjing, 210000 Jiangsu China

**Keywords:** Ecotype, Genetic differentiation, Local adaptationMorphological divergence, *Phrynocephalus*, Qinghai-Tibetan plateau

## Abstract

**Background:**

The Qinghai-Tibetan Plateau (QTP) is the world’s highest and largest plateau, but the role of its uplift in the evolution of species or biotas still remains poorly known. Toad-headed lizards of the reproductively bimodal genus *Phrynocephalus* are a clade of agamids, with all viviparous species restricted to the QTP and adjacent regions. The eastern part of the range of the viviparous taxa is occupied by three closely related but taxonomically controversial species, *P. guinanensis*, *P. putjatia* and *P. vlangalii*. Here, we combined genetic (mitochondrial ND4 gene and nine microsatellite loci), morphological (11 mensural and 11 meristic variables), and ecological (nine climatic variables) data to explore possible scenarios that may explain the discordance between genetic and morphological patterns, and to test whether morphological divergence is associated with local adaptation.

**Results:**

We found weak genetic differentiation but pronounced morphological divergence, especially between *P. guinanensis* and *P. vlangalii*. Genetically, the species boundary was not so clear between any species pair. Morphologically, the species boundary was clear between *P. guinanensis* and *P. vlangalii* but not between other two species pairs. Body size and scale characters accounted best for morphological divergence between species. Morphological divergence was related to habitat types that differ climatically.

**Conclusions:**

Our study provides evidence for genetic and morphological divergence among the three closely related viviparous species of *Phrynocephalus* lizards, and supports the idea that natural selection in spatially heterogeneous environments can lead to population divergence even in the presence of gene flow. Our study supports the hypothesis that the evolutionary divergence between viviparous *Phrynocephalus* species was a consequence of environmental change after the uplift of the QTP.

**Electronic supplementary material:**

The online version of this article (10.1186/s12862-019-1443-y) contains supplementary material, which is available to authorized users.

## Background

Genetic divergence and speciation can occur in different parts of an ancestral species’ range and even within habitats [1]. Genetic divergence within and among species is not always accompanied by clear phenotypic (morphological, anatomical, physiological, and/or behavioral) differences due to silent mutations or phenotypic convergence [2]. However, it can give rise to significant phenotypic changes due to novel adaptations via selection that drives local adaptation [2]. Depending on its relationship to the environment, phenotypic variation may be either adaptive or non-adaptive. Adaptive phenotypic variation often occurs between populations that live in different environments and is associated with local adaptation [1, 3]. Phenotype-environment correlations have been documented in a wide variety of taxa from plant [4] to invertebrates [5, 6] and vertebrates [7–9], particularly with respect to the morphology-environment correlation. Functionally important morphological traits that are highly associated with reproductive success, heat exchange, water transfer and locomotion are particularly suitable to studies of speciation and population evolution [10].

Integrative analyses that combine molecular phylogeny, phylogenetic biogeography and phenotypic evolution represent a powerful approach to identify divergent clades with or without phenotypic differentiation, to detect population genetic structure, and to assess early stages of the speciation process [5, 11, 12]. Studies on lizards have showed that use of different habitats may lead to divergent selection on traits that define body size, body shape, coloration pattern and/or scale characteristics (size, number and scutellation), resulting in morphological diversification among populations or species [13–15]. However, to date, few studies have used an integrative approach to address morphological and species diversification of lizards in the Old World.

**Fig. 1:**
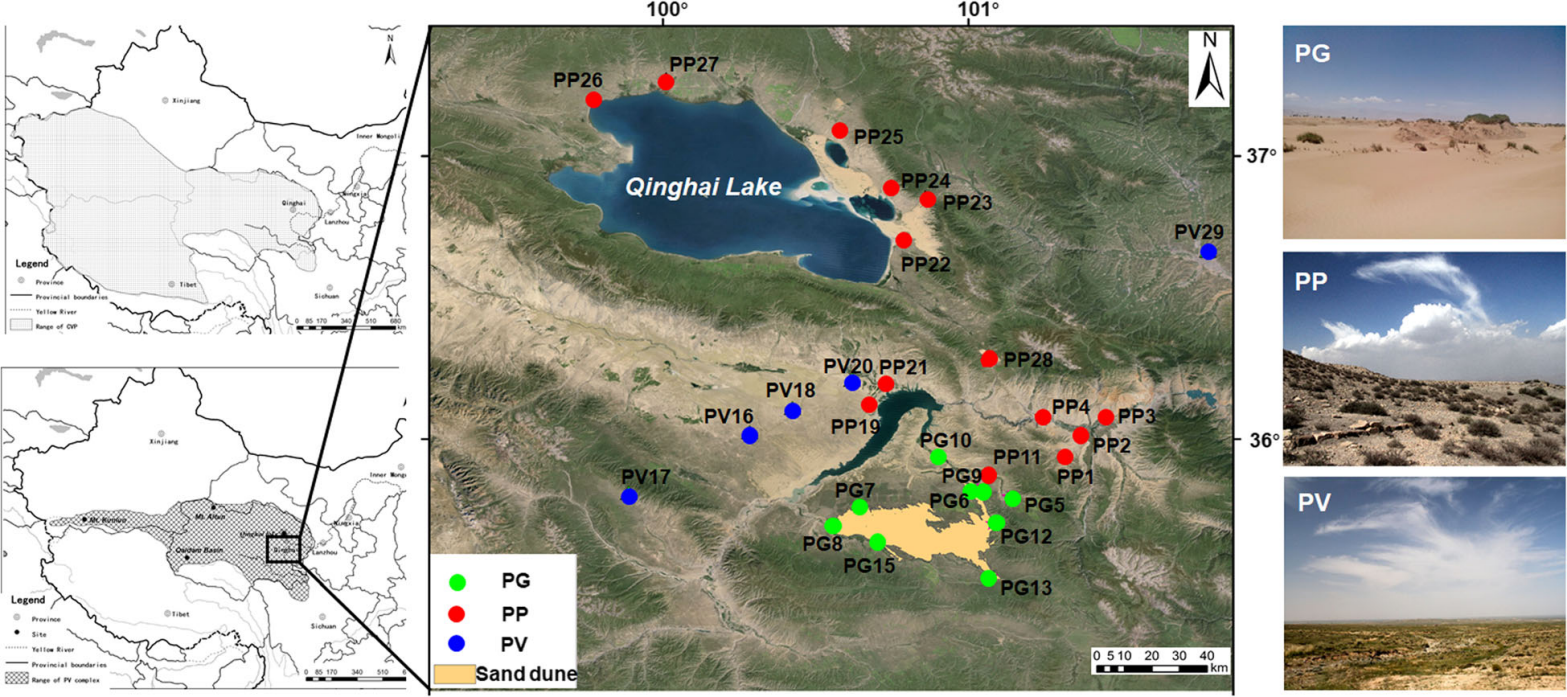
Map of mainland China [upper-left, showing the distribution (shaded area) of six viviparous *Phrynocephalus* species], map of northwestern China [lower-left, showing the distribution (shaded area) of the species studied herein], sampling localities (middle), and typical habitats used by *P. guinanensis* (PG, upper-right), *P. putjatia* (PP, middle-right), and *P. vlangalii* (PV, lower-right). See Table 1 for detailed information on sampling localities

Toad-headed lizards of the reproductively bimodal genus *Phrynocephalus* (Agamidae) inhabit desert, arid and semiarid regions in Central and West Asia and North-Northwest China, with all viviparous species restricted to the Qinghai-Tibetan Plateau (QTP) and adjacent regions (Fig. 1) [16]. The eastern part of the range of the viviparous taxa is occupied by a group of three closely related but taxonomically controversial species, *P. guinanensis*, *P. putjatia* and *P. vlangalii* [17–20]. *Phrynocephalus vlangalii* is the most widespread species and inhabits arid and semiarid habitats in the western part of the group’s range across an altitudinal range from 2200 to 4500 m, *P. putjatia* is the oldest species restricted to steppe desert habitats at relatively low altitudes (2200–3300 m) around Qinghai Lake, and *P. guinanensis* is the most narrowly distributed species restricted to sand dunes (2700–3500 m) in the south of Qinghai Lake (Fig. 1) [17, 19–22]. The ranges of *P. putjatia* and *P. vlangalii* overlap around Qinghai Lake, but neither occurs in the range of *P. guinanensis* [17, 20]. Morphological data support a valid species of *P. guinanensis*, but genetic data do not support that distinction [17, 19]. So the currently accepted status of *P. guinanensis* is an ecotype of *P. putjatia* [19].

However, as the ecotype hypothesis has yet to be empirically tested, a knowledge gap remains. In order to fill the gap we collected specimens from 28 localities (Fig. 1), we downloaded climatic data form WorldClim and trimmed to each sampling locality, took morphological measurements and used molecular markers (mitochondrial ND4 gene and nine microsatellite loci) to assess the structure and clustering of specimens. We then calculated distances based on all of them and compared the dissimilarity matrices. We aim to explore possible scenarios that may explain the discordance between genetic and morphological patterns, and to test whether morphological divergence among these species is associated with local adaptation. We predict that, if the ecotype hypothesis were true, the morphology should be well correlated with climate, or at least more than with genetics.

## Results

### Genetic polymorphism

We obtained a sequence of 684 base pairs (bp) of the mitochondrial ND4 gene, which contained nine singleton variable sites and 133 parsimony informative sites. Eight haplotypes were shared by two species (four by PG and PP, two by PP and PV, and two by PG and PV), and only one haplotype was shared by all three species (Additional file 5: Figure S1). Within individual localities, haplotype diversity varied from 0 to 0.82, and nucleotide diversity from 0 to 0.052 (Table 1). Haplotype diversity (*h* ± SD) was 0.92 ± 0.01 in PG, 0.94 ± 0.01 in PP*,* and 0.86 ± 0.02 in PV; nucleotide diversity [(π ± SD) × 10^3^] was 7.48 ± 4.04 in PG, 23.07 ± 11.44 in PP and 64.23 ± 31.19 in PV (Table 1).

**Table 1:** Sampling locality information, genetic diversity and demographic statistics for partial ND4 sequences

Locality	*N*	Longitude (°)	Latitude (°)	Elevation (m)	Nhap	Haplotype diversity (*h* ± SD)	Nucleotide diversity (*π* ± SD) × 10^3^	Tajima’s *D*	Fu’s *Fs*	SSD	Rag
PG05	16	101.13	35.80	3191	5	0.71 ± 0.09	15.17 ± 11.45	−2.37^***^	6.84	0.01	0.03
PG06	16	101.03	35.82	3116	3	0.61 ± 0.09	1.11 ± 0.22	0.66	0.42	0.01	0.12
PG07	16	100.64	35.76	3248	1	–	–	–	–	–	–
PG08	11	100.56	35.70	3207	2	0.44 ± 0.13	1.28 ± 0.39	0.85	2.01	0.16	0.70
PG09	16	101.02	35.82	3126	6	0.78 ± 0.07	4.71 ± 0.60	0.26	0.61	0.06	0.12
PG10	16	100.90	35.94	2675	3	0.43 ± 0.13	5.15 ± 1.48	1.10	5.00	0.15	0.44
PG12	16	101.08	35.70	3509	5	0.53 ± 0.14	1.18 ± 0.43	−1.03	−1.98^*^	0.01	0.11
PG13	22	101.07	35.51	3340	6	0.77 ± 0.06	5.42 ± 0.37	1.20	1.83	0.09	0.16
PG15	16	100.70	35.64	2995	6	0.82 ± 0.07	3.86 ± 0.93	−0.46	0.06	0.29^*^	0.05
PP01	15	101.45	36.08	2273	2	0.13 ± 0.11	0.19 ± 0.16	−1.16	−0.65	0.04	0.56
PP02	15	101.37	36.01	2278	2	0.42 ± 0.11	6.13 ± 1.65	1.38	8.29	0.24^*^	0.69
PP03	16	101.24	36.08	2259	2	0.53 ± 0.06	6.91 ± 0.72	2.73	9.28	0.55^***^	0.78
PP04	16	101.32	35.94	2578	1	–	–	–	–	–	–
PP11	16	101.06	35.87	2930	5	0.77 ± 0.07	5.77 ± 1.23	0.72	2.31	0.07	0.18
PP19	16	100.68	36.13	2703	4	0.52 ± 0.13	1.50 ± 0.61	−1.48	−0.21	0.01	0.09
PP21	11	100.73	36.20	2602	3	0.35 ± 0.17	2.29 ± 1.16	−1.40	1.55	0.11	0.49
PP22	16	100.79	36.70	3228	4	0.35 ± 0.15	16.67 ± 11.22	−2.24^***^	9.71	0.08	0.49
PP23	16	100.86	36.85	3237	3	0.50 ± 0.12	1.40 ± 0.68	−1.20	0.90	0.02	0.17
PP24	15	100.75	36.89	3307	3	0.51 ± 0.12	19.88 ± 3.89	2.00	13.42	0.42^***^	0.55
PP25	5	100.58	37.09	3255	3	0.70 ± 0.22	15.79 ± 8.96	−1.24	3.98	0.14	0.23
PP26	16	99.78	37.20	3251	3	0.49 ± 0.12	1.40 ± 0.68	−1.20	0.90	0.02	0.17
PP27	14	100.01	37.27	3247	5	0.66 ± 0.12	1.54 ± 0.45	−1.14	−1.50	0.004	0.05
PP28	16	101.06	36.28	3354	3	0.51 ± 0.13	0.82 ± 0.24	−0.19	−0.18	0.02	0.17
PV16	16	100.28	36.03	3100	2	0.13 ± 0.11	0.37 ± 0.31	−1.50^*^	0.18	0.02	0.80
PV17	16	99.89	35.81	3700	5	0.72 ± 0.10	1.43 ± 0.30	−0.60	−1.49	0.02	0.16
PV18	15	100.42	36.11	2987	4	0.62 ± 0.12	1.06 ± 0.27	−0.64	−1.04	0.03	0.20
PV20	16	100.62	36.21	2793	2	0.13 ± 0.11	0.18 ± 0.16	−1.16	−0.70	0.04	0.58
PV29	15	101.78	36.67	3200	6	0.76 ± 0.10	52.49 ± 10.15	2.08	12.55	0.14	0.14
Total	426				67	0.96 ± 0.01	34.43 ± 16.77	0.30	0.79	0.01	0.004
PG	145				29	0.92 ± 0.01	7.48 ± 4.04	−2.19	−6.17	0.003	0.01
PP	203				30	0.94 ± 0.01	23.07 ± 11.44	−0.51	5.07	0.02	0.01^*^
PV	78				18	0.86 ± 0.02	64.23 ± 31.19	3.53	27.03	0.05^***^	0.04^***^

*N* sample size (number of individuals), *Nhap* number of haplotypes, *Fu’s Fs* statistics of Fu’s *Fs* test, *Tajima’s D* statistics of Tajima’s *D* test, *SSD* sum of square deviation, *Rag* Harpending’s raggedness index. *PG P. guinanensis*, *PP P. putjatia*, *PV P. vlangalii*

^*^ *P* < 0.05; ^***^ *P* < 0.001

A total of 462 lizards were genotyped and scored at nine microsatellite loci. The number of alleles per locus varied from 14 to 60, with a mean of 37. The mean observed heterozygosity was 0.582, and the mean expected heterozygosity was 0.916 (Additional file 2: Table S2).

### Relationships among mtDNA haplotypes

**Fig. 2:**
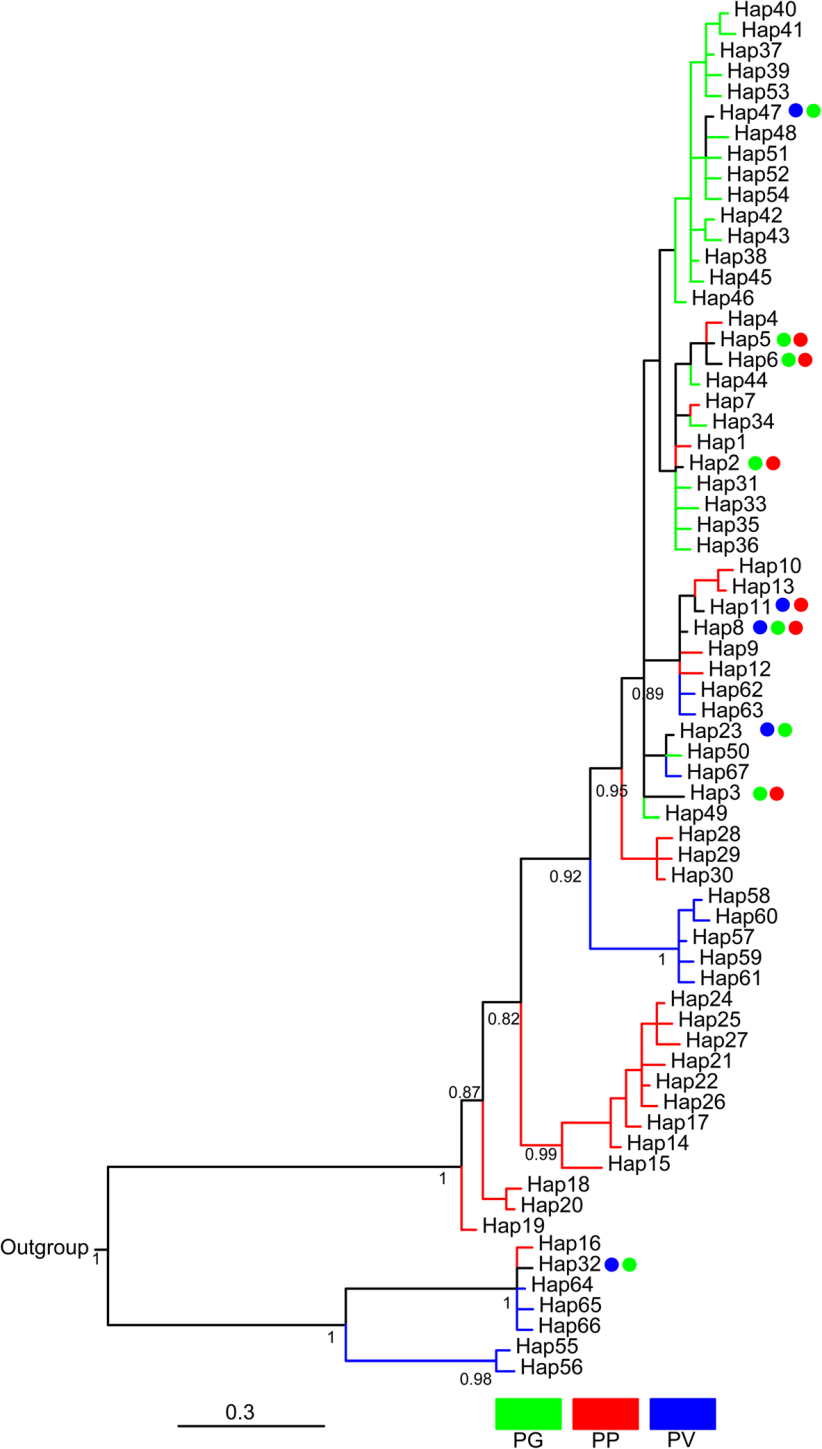
A Bayesian 50% concensus phylogenetic tree based on ND4 haplotypes. Numbers under the tree branches are posterior probabilities

A clade of PV included individuals from PV16, PV20 and PV29; individuals from PV17 formed a clade; individuals from PV18 were admixed in branch. Because of low support values at several nodes, there was a polytomy consisting of PP, PV17, and all other groups of the admixed section, within which a clade of PP included individuals from seven localities (PP22–27) northeast of Qinghai Lake, PV17 and the remaining haplotypes of the three species. Approximately 89% (25/28) of PG haplotypes formed a PG subclade (Fig. 2). Median-joining network based on ND4 haplotypes showed similar grouping patterns to the gene tree and all clades and subclades were recovered (Additional file 5: Figure S1). The mean pairwise distance was 2.0% between PG and PP, 7.1% between PG and PV, and 7.3% between PP and PV. The mean pairwise distance within species was 0.8% for PG, 2.4% for PP, and 7.1% for PV.

### Population structure

**Fig. 3:**
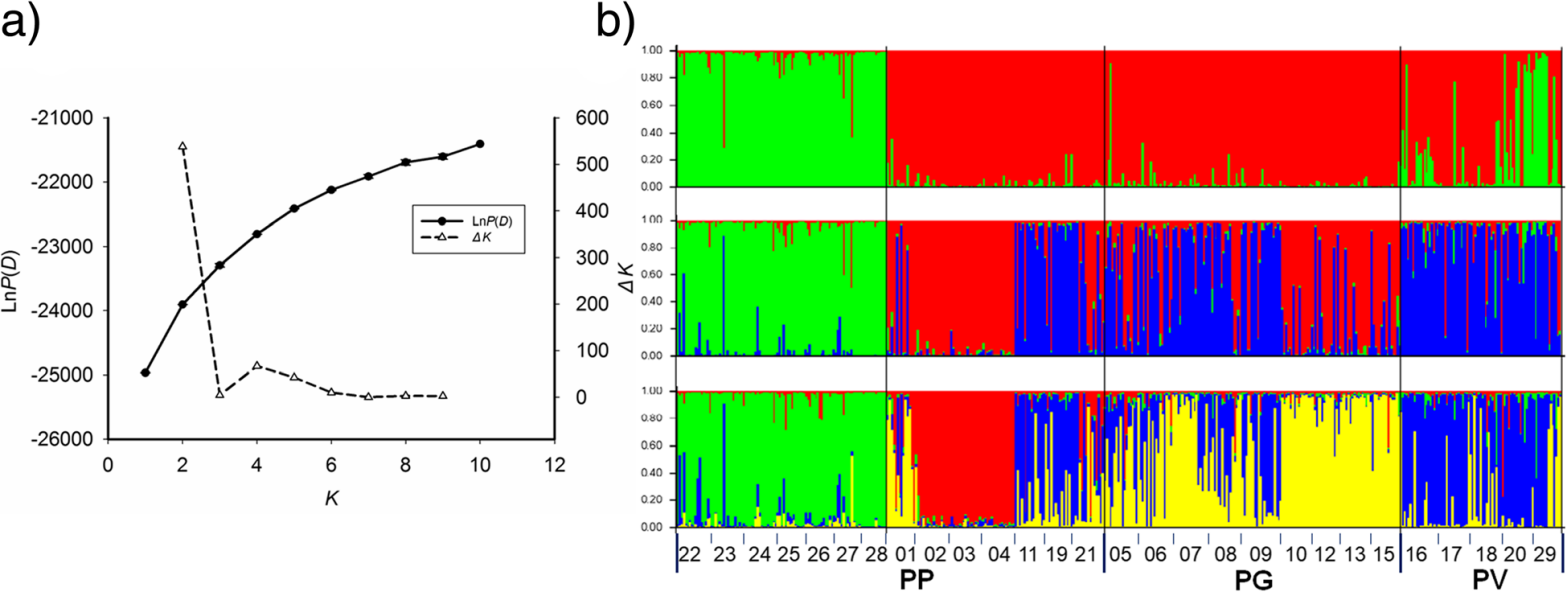
Results of Bayesian model-based clustering in STRUCTURE based on nine microsatellite markers. **a** The plot of the mean posterior probability Ln*P*(*D*) and values of *ΔK* against *K* values (number of clusters) resulting from 10 runs. **b** Bar plots showing Bayesian assignment probabilities from the software STRUCTURE 2.3.2 for two, three, and four clusters. Each bar represents an individual and its probabilities of being assigned to clusters. See Fig. 1 for definitions for PG, PP and PV

Assignment tests based on nine microsatellite loci identified two distinct genetic clusters (Fig. 3a). One (red) groups individuals from all three species together, and the other (green) groups individuals from PP and PV. Two major genetic clusters were revealed in PP with one including individuals from localities northeast of Qinghai Lake, and the other including individuals mostly from localities south of the lake. Individuals of PG showed a pure genetic cluster, while individuals of PV had admixed assignment (Fig. 3b). At larger values (3–4) of *K*, additional clusters appeared. When STRUCTURE was run under the assumption that the data represented three separate populations (*K* = 3), individuals from localities south of Qinghai Lake were still assigned to their respective clusters (green), but individuals from localities south of the lake and PG individuals were assigned to two groups with moderate probability (red and blue), PV individuals were assigned to a distinct, third cluster (blue) (Fig. 3b).

### Morphological divergence

**Fig. 4:**
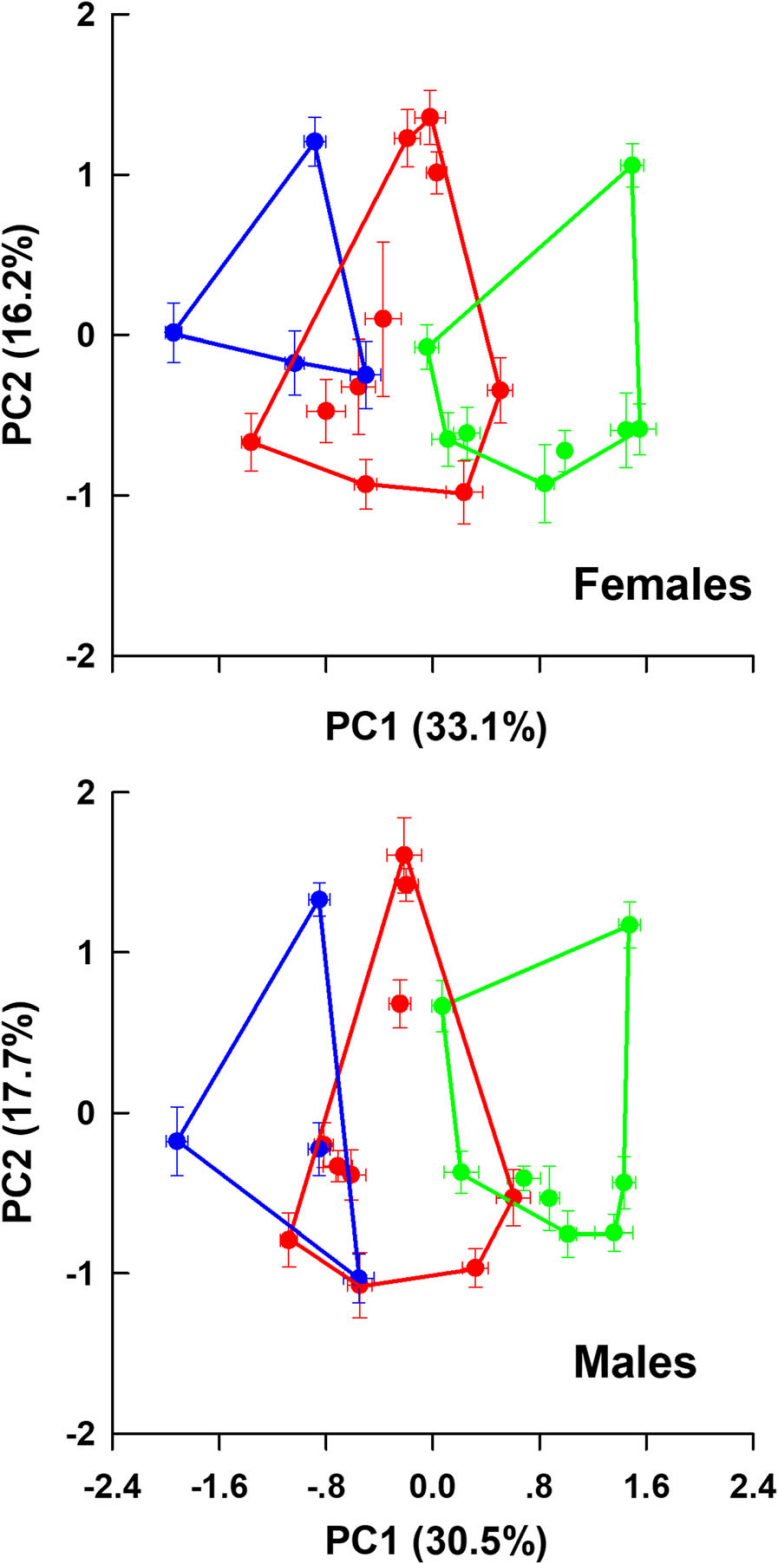
Positions of the species studied herein in a two-dimension space defined by the first two axes of a principal component analysis based on 22 adult morphological variables. Size effects were removed when necessary using residuals from the regressions of the corresponding variables on SVL. Mean values (±SE) for factor scores on the first two axes of the populations measured are given in the figure. Green dots and lines: *P. guinanensis*; red dots and lines: *P. putjatia*; blue dots and lines: *P. vlangalii*

All examined morphological variables except tail length differed among the three species (Table 2). All 11 mensural variables differed between the sexes; only two (superciliaris and dorsal scales) of 11 meristic variables differed between the sexes (Table 2). PCAs on the body dimensions and scalation characters performed separately for each sex showed that mean scores on the first two axes differed among the three species in both sexes (Additional file 3: Table S3, Additional file 6: Figure S2). Mean scores on the first axis differed among the three species, and in both sexes mean scores were greatest in PG and smallest in PV (Table 3, Fig. 4). Differences were also found between mean scores on the second axis in females, with mean scores being greatest in PV and smallest in PG (Table 3, Fig. 4).

**Table 2:** Mensural and meristic data, expressed as mean ± SE and range, for adults of the species studied herein

	P. guinanensis		P. putjatia		P. *vlangalii*		Effects		
	Females	Males	Females	Males	Females	Males	Species	Sex	Species × Sex
N	89	86	95	100	54	52			
Snout-vent length (mm)	66.7 ± 0.6	66.1 ± 0.7	61.0 ± 0.5	60.4 ± 0.4	65.7 ± 1.0	63.5 ± 0.8	F_2, 470_ = 54.79, *P* < 0.0001; PG^a^, PP^c^, PV^b^	F_1, 470_ = 4.74, *P* = 0.030; F > M	F_2, 470_ = 0.98, *P* = 0.378
	53.2–77.1	51.0–78.8	49.3–70.5	50.0–69.8	51.5–83.4	52.7–77.2			
Head length (mm)	16.8 ± 0.1	17.2 ± 0.1	15.2 ± 0.1	15.5 ± 0.1	15.9 ± 0.2	15.9 ± 0.2	F_2, 469_ = 57.15, *P* < 0.0001; PG^a^, PP^b^, PV^b^	F_1, 469_ = 50.83, *P* < 0.0001; F < M	F_2, 469_ = 0.95, *P* = 0.388
	14.5–19.1	13.8–19.9	13.5–16.9	13.5–17.9	12.7–18.8	13.3–18.1			
Head width (mm)	13.8 ± 0.1	14.2 ± 0.1	12.8 ± 0.1	13.0 ± 0.1	13.8 ± 0.2	13.9 ± 0.2	F_2, 469_ = 14.05, *P* < 0.0001; PG^a^, PP^b^, PV^a^	F_1, 469_ = 43.74, *P* < 0.0001; F < M	F_2, 469_ = 2.16, *P* = 0.116
	11.9–15.6	11.2–16.5	11.1–14.6	11.2–14.7	11.0–16.4	11.8–16.2			
Abdomen length (mm)	36.0 ± 0.3	34.0 ± 0.4	33.2 ± 0.3	31.4 ± 0.3	36.8 ± 0.6	33.4 ± 0.5	F_2, 469_ = 13.15, *P* < 0.0001; PG^c^, PP^b^, PV^a^	F_1, 469_ = 125.32, *P* < 0.0001; F > M	F_2, 469_ = 1.23, *P* = 0.292
	28.0–42.7	26.1–43.0	26.2–40.7	24.8–38.2	27.0–46.9	27.0–41.0			
Tail length (mm)	60.0 ± 0.7	65.5 ± 0.8	54.6 ± 0.7	59.5 ± 0.7	58.0 ± 0.9	63.0 ± 0.9	F_2, 469_ = 1.73, *P* = 0.179	F_1, 469_ = 138.98, *P* < 0.0001; F < M	F_2, 429_ = 0.68, *P* = 0.506
	46.9–76.0	50.8–81.0	41.4–66.8	42.9–71.7	44.2–70.2	47.6–75.5			
Fore-limb length (mm)	18.3 ± 0.2	18.9 ± 0.3	16.7 ± 0.1	16.9 ± 0.1	18.0 ± 0.2	18.0 ± 0.2	F_2, 469_ = 4.68, *P* < 0.01; PG^a^, PP^b^, PV^ab^	F_1, 469_ = 19.27, *P* < 0.0001; F < M	F_2, 469_ = 1.19, *P* = 0.306
	14.8–23.5	15.3–24.8	14.1–19.0	14.2–18.8	14.6–21.6	14.4–21.2			
Hind-limb length (mm)	28.5 ± 0.3	30.1 ± 0.3	26.0 ± 0.2	27.3 ± 0.2	26.7 ± 0.3	27.7 ± 0.3	F_2, 469_ = 31.75, *P* < 0.0001; PG^a^, PP^b^, PV^c^	F_1, 469_ = 117.23, *P* < 0.0001; F < M	F_2, 469_ = 0.62, *P* = 0.540
	23.2–34.9	24.0–35.6	22.1–30.6	21.7–31.1	21.4–31.7	23.1–31.6			
4th finger length (mm)	5.7 ± 0.06	5.9 ± 0.06	5.1 ± 0.04	5.3 ± 0.04	5.3 ± 0.1	5.6 ± 0.1	F_2, 469_ = 20.86, *P* < 0.0001; PG^a^, PP^b^, PV^b^	F_1, 469_ = 38.20, P < 0.0001; F < M	F_2, 469_ = 1.80, *P* = 0.166
	4.6–6.9	4.7–7.3	4.2–6.0	4.4–6.2	4.3–6.7	4.5–7.1			
4th toe length (mm)	8.9 ± 0.1	9.5 ± 0.1	7.9 ± 0.05	8.3 ± 0.06	7.6 ± 0.1	8.1 ± 0.1	F_2, 469_ = 98.06, *P* < 0.0001; PG^a^, PP^b^, PV^c^	F_1, 469_ = 76.41, P < 0.0001; F < M	F_2, 469_ = 1.37, *P* = 0.255
	7.3–10.7	7.2–13.2	6.6–9.0	7.2–10.3	6.1–9.5	6.3–9.3			
Claw length of the 4th finger (mm)	2.6 ± 0.04	2.3 ± 0.04	2.4 ± 0.03	2.3 ± 0.03	2.0 ± 0.04	1.9 ± 0.04	F_2, 469_ = 73.94, *P* < 0.0001; PG^a^, PP^a^, PV^b^	F_1, 469_ = 8.33, *P* < 0.005; F > M	F_2, 469_ = 3.34, *P* = 0.036
	1.8–3.6	1.6–3.4	1.6–3.1	1.4–3.2	1.3–2.7	1.5–2.9			
Claw length of the 4th toe (mm)	2.3 ± 0.04	2.3 ± 0.03	2.1 ± 0.03	2.2 ± 0.03	1.9 ± 0.03	2.0 ± 0.04	F_2, 469_ = 40.67, *P* < 0.0001; PG^a^, PP^b^, PV^c^	F_1, 469_ = 4.92, *P* < 0.027; F < M	F_2, 469_ = 0.71, *P* = 0.491
	1.1–3.2	1.7–3.1	1.4–2.8	1.4–3.2	1.5–2.4	1.5–3.0			
Nasal scales	4.9 ± 0.1	5.0 ± 0.1	4.0 ± 0.06	4.0 ± 0.06	4.0 ± 0.08	3.9 ± 0.08	F_2, 470_ = 97.22, *P* < 0.0001; PG^a^, PP^b^, PV^b^	F_1, 470_ = 0.17, *P* = 0.680	F_2, 470_ = 0.12, *P* = 0.888
	3–8	3–8	3–5	3–5	3–5	3–5			
Internasal scales	6.9 ± 0.1	6.8 ± 0.1	5.5 ± 0.07	5.5 ± 0.07	5.5 ± 0.1	5.6 ± 0.1	F_2, 470_ = 147.54, *P* < 0.0001; PG^a^, PP^b^, PV^b^	F_1, 470_ = 0.08, *P* = 0.779	F_2, 470_ = 0.28, *P* = 0.755
	5–8	5–8	5–7	5–7	4–7	4–7			
Scales around parietal eye	9.8 ± 0.1	9.8 ± 0.1	9.1 ± 0.1	9.1 ± 0.1	7.8 ± 0.1	7.8 ± 0.2	F_2, 470_ = 129.78, *P* < 0.0001; PG^a^, PP^b^, PV^c^	F_1, 470_ = 0.01, *P* = 0.913	F_2, 470_ = 0.01, *P* = 0.991
	8–12	8–12	6–12	7–11	6–10	5–11			
Supraocular scales	15.8 ± 0.2	15.7 ± 0.2	13.5 ± 0.2	13.3 ± 0.2	13.1 ± 0.2	12.7 ± 0.2	F_2, 470_ = 119.30, *P* < 0.0001; PG^a^, PP^b^, PV^c^	F_1, 470_ = 1.73, *P* = 0.190	F_2, 470_ = 0.40, *P* = 0.669
	12–21	11–20	10–19	10–19	10–18	10–16			
Superciliaris	11.4 ± 0.1	11.2 ± 0.1	10.9 ± 0.1	10.9 ± 0.1	11.2 ± 0.1	10.6 ± 0.1	F_2, 470_ = 8.51, *P* < 0.0003; PG^a^, PP^b^, PV^b^	F_1, 430_ = 9.415, *P* < 0.003; F > M	F_2, 430_ = 1.62, *P* = 0.200
	9–14	9–14	9–13	8–13	9–14	9–12			
Gular folds	23.6 ± 0.3	23.2 ± 0.3	22.7 ± 0.3	21.8 ± 0.3	21.3 ± 0.3	21.0 ± 0.3	F_2, 470_ = 27.14, *P* < 0.0001; PG^a^, PP^b^, PV^c^	F_1, 470_ = 3.72, *P* = 0.054	F_2, 470_ = 0.58, *P* = 0.559
	17–28	16–31	16–30	17–28	17–26	16–25			
Dorsal scales	134.5 ± 1.4	131.4 ± 1.3	117.6 ± 1.5	115.5 ± 1.2	105.2 ± 1.6	101.6 ± 1.5	F_2, 470_ = 193.22, *P* < 0.0001; PG^a^, PP^b^, PV^c^	F_1, 430_ = 6.16, *P* = 0.013; F > M	F_2, 430_ = 0.13, *P* = 0.882
	100–162	107–158	91–149	94–154	78–127	79–118			
Ventral scales	115.3 ± 1.2	116.7 ± 1.3	100.5 ± 1.2	101.7 ± 1.1	86.6 ± 1.4	87.1 ± 1.4	F_2, 470_ = 229.86, *P* < 0.0001; PG^a^, PP^b^, PV^c^	F_1, 430_ = 0.92, *P* = 0.338	F_2, 430_ = 0.05, *P* = 0.951
	89–138	83–153	74–129	81–131	71–112	73–118			
Subdigital lamellae of the 4th finger	16.9 ± 0.2	17.1 ± 0.1	15.6 ± 0.2	15.3 ± 0.2	14.3 ± 0.2	14.2 ± 0.2	F_2, 470_ = 113.47, *P* < 0.0001; PG^a^, PP^b^, PV^c^	F_1, 470_ = 0.17, *P* = 0.683	F_2, 470_ = 1.48, *P* = 0.229
	13–21	14–22	11–19	11–19	12–18	11–18			
Subdigital lamellae of the 4th toe	25.3 ± 0.2	25.5 ± 0.2	22.8 ± 0.2	22.5 ± 0.2	18.6 ± 0.3	18.4 ± 0.2	F_2, 470_ = 398.62, *P* < 0.0001; PG^a^, PP^b^, PV^c^	F_1, 470_ = 0.14, *P* = 0.712	F_2, 470_ = 0.75, *P* = 0.472
	21–30	21–31	16–27	18–27	15–23	15–22			
Scales around mid-body	159.0 ± 1.7	161.6 ± 1.6	141.2 ± 2.1	143.5 ± 2.2	135.0 ± 2.4	135.9 ± 2.0	F_2, 470_ = 75.52, *P* < 0.0001; PG^a^, PP^b^, PV^c^	F_1, 470_ = 1.28, *P* = 0.258	F_2, 470_ = 0.07, *P* = 0.928
	127–203	121–217	102–201	106–204	108–202	111–173			

Data are analyzed using two-way ANOVA (SVL and meristic variables) or two-way ANCOVA (mensural variables other than SVL) with SVL as the covariate and species and sex as the factors

*PG* P. guinanensis, *PP* P. putjatia, *PV* P. *vlangalii*, *F* females, and *M* males

**Table 3:** Loading of the first two axes of a principal component (PC) analysis on 22 adult morphological variables

	Females		Males	
	PC1	PC2	PC1	PC2
Snout-vent length	0.305	− 0.245	0.485	− 0.249
Head length	0.606	0.451	0.504	0.479
Head width	0.122	0.664	0.208	0.601
Abdomen length	−0.274	0.120	−0.145	0.070
Tail length	0.371	0.670	0.199	0.684
Fore-limb length	0.341	**0.780**	0.215	**0.802**
Hind-limb length	0.623	0.593	0.478	0.669
4th finger length	0.588	0.578	0.373	**0.754**
4th toe length	**0.759**	0.397	0.618	0.529
Claw length of the 4th finger	0.471	−0.380	0.338	−0.303
Claw length of the 4th toe	0.371	−0.354	0.306	−0.329
Nasal scales	0.661	0.012	0.636	0.038
Internasal scales	0.619	−0.001	0.599	0.071
Scales around parietal eye	0.593	−0.079	0.580	−0.130
Supraocular scales	0.630	−0.300	**0.733**	−0.286
Superciliaris	0.339	−0.235	0.425	−0.336
Gular folds	0.393	−0.005	0.355	0.052
Dorsal scales	**0.797**	−0.350	**0.843**	−0.238
Ventral scales	**0.805**	−0.391	**0.816**	−0.338
Subdigital lamellae of the 4th finger	**0.760**	−0.110	**0.800**	−0.021
Subdigital lamellae of the 4th toe	**0.829**	−0.244	**0.836**	−0.128
Scales around midbody	0.646	−0.424	0.673	−0.483
Variance explained (%)	33.1	16.2	30.5	17.7
Factor scores on PC1	*F*_2, 235_ = 164.34, *P* < 0.001 PG^a^, PP^b^, PV^c^		*F*_2, 235_ = 215.60, *P* < 0.001 PG^a^, NPP^c^, SPP^b^, PV^c^	
Factor scores on PC2	*F*_2, 235_ = 5.86, *P* < 0.01 PG^b^, PP^ab^, PV^a^		*F*_2, 235_ = 2.38, *P* = 0.095	

Size effects are removed in all cases by using residuals from regressions on SVL. Variables with the main contribution to each factor are in bold. Species with different superscripts differ significantly (Tukey’s test, α = 0.05; a > b > c). See Fig. 1 for definitions for PG, PP and PV

### Climatic differences

**Fig. 5:**
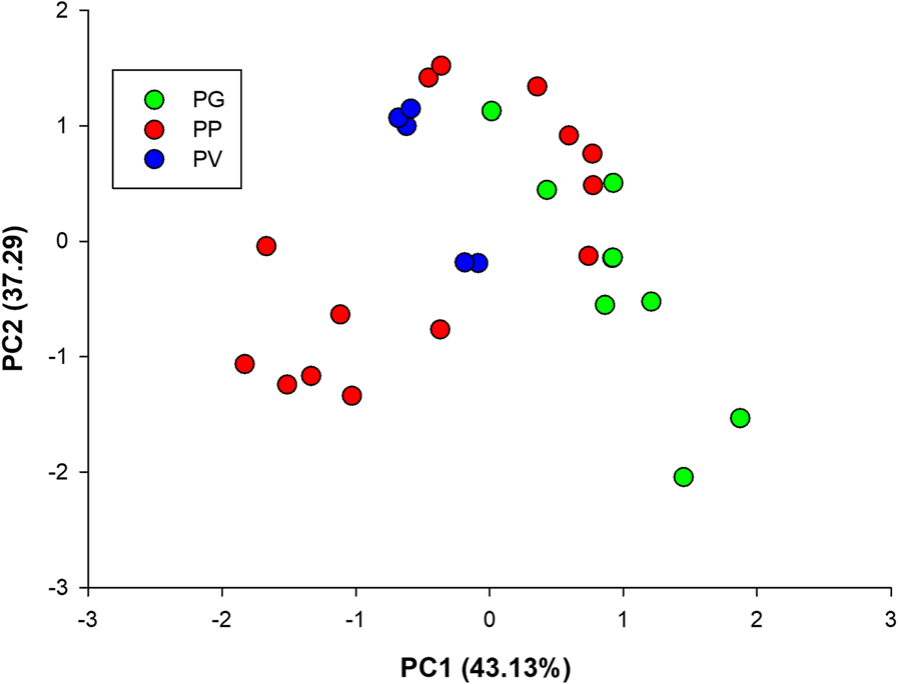
Positions of the species studied herein in a two-dimension space defined by the first two axes of a principal component analysis based on nine climatic variables

A PCA of nine climatic variables for 28 localities revealed that the first two components accounted for 80% of the variance (Additional file 4: Table S4). Mean PC scores on the first axis (*F*_2, 25_ = 10.20, *P* < 0.001; PG^a^, PP^b^, PV^b^) differed significantly among the three species, while mean PC scores on the second axis did not (*F*_2, 25_ = 1.29, *P* = 0.29). Overall, climatic differences were more evident between PP and PG than between any other pairs of species (Fig. 5).

### Distance correlation analysis

**Fig. 6:**
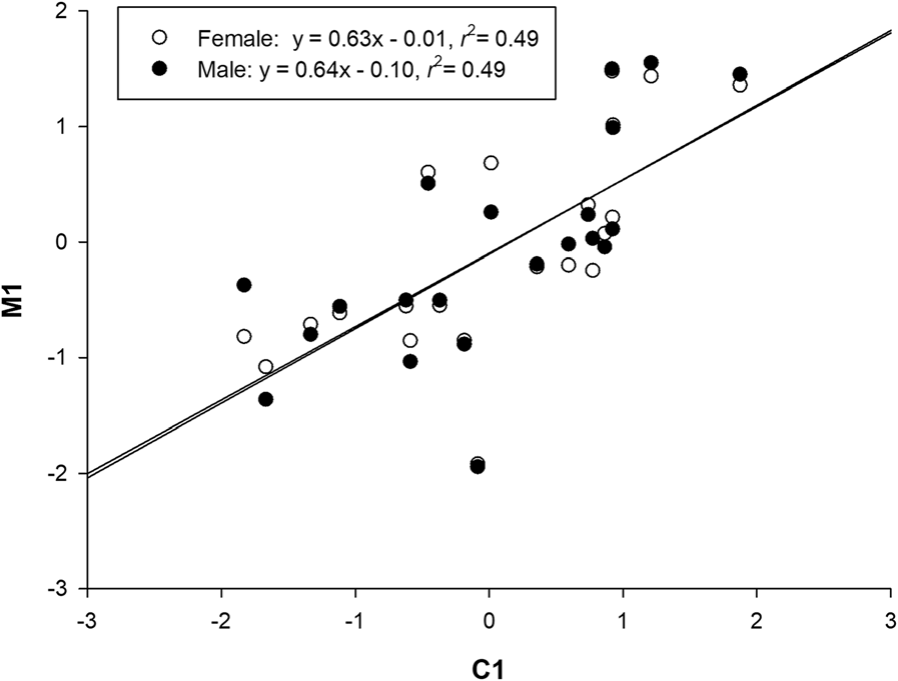
Relationship between mean scores of the first morphology PC axis (M1) and the first climate PC axis (C1)

The first morphology PC axis (M1) was positively related to the first climate PC axis (C1) in males (*F*_1, 19_ = 18.22, *P* < 0.001), and so was in females (*F*_1, 19_ = 18.24, *P* < 0.001) (Fig. 6). The single Mantel tests for the combined data matrix showed that: (1) geographic distance was significantly related to C1 and M1 in both sexes, and to genetic distance inferred from the ND4 gene; and (2) the first climate PC axis was significantly related to the first morphology PC axis in both sexes (Table 4). In both sexes M1 was significantly related to genetic distance inferred from the ND4 gene. Morphological divergence and genetic distance were spatially patterned and both were climatically dependent (Table 4). Holding C1 constant with the partial Mantel test, we found that the coefficient of a correlation between morphological divergence and genetic distance was 0.187 for males and 0.176 for females, and in both sexes the correlation was not statistically significant (Table 4). Holding geographic distance constant, we found once again that C1 significantly correlated with M1 in both sexes (Table 4).

**Table 4:** Results of single and partial Mantel tests on populations of the species studied herein, showing the correlation between two matrices

Single Mantel test	*r*	*P* value	Partial Mantel test	*r*	*P* value
Geographic distance vs Climate PC1	**0.691**	**0.001**	Holding geographic distance constant		
Geographic distance vs Climate PC2	−0.029	0.557	Climate PC1 vs Male morphology PC1	**0.254**	**0.019**
Geographic distance vs Male morphology PC1	**0.294**	**0.022**	Climate PC1 vs Male morphology PC2	−0.187	0.999
Geographic distance vs Male morphology PC2	0.006	0.439	Climate PC1 vs Female morphology PC1	**0.237**	**0.021**
Geographic distance vs Female morphology PC1	**0.303**	**0.012**	Climate PC1 vs Female morphology PC2	−0.171	0.988
Geographic distance vs Female morphology PC2	0.005	0.445	Climate PC1 vs Genetic distance 1	0.130	0.139
Geographic distance vs Genetic distance 1	**0.388**	**0.006**	Climate PC1 vs Genetic distance 2	0.097	0.252
Geographic distance vs Genetic distance 2	0.192	0.121	Holding climate PC1 constant		
Climate PC1 vs Male morphology PC1	**0.379**	**0.001**	Genetic distance 1 vs Male morphology PC1	0.187	0.106
Climate PC1 vs Male morphology PC2	−0.131	0.928	Genetic distance 1 vs Female morphology PC1	0.176	0.115
Climate PC1 vs Female morphology PC1	**0.371**	**0.002**			
Climate PC1 vs Female morphology PC2	−0.120	0.894			
Climate PC1 vs Genetic distance 1	**0.355**	**0.003**			
Climate PC1 vs Genetic distance 2	0.201	0.065			
Male morphology PC1 vs genetic distance 1	**0.296**	**0.012**			
Male morphology PC2 vs genetic distance 1	−0.112	0.842			
Female morphology PC1 vs genetic distance 1	**0.284**	**0.009**			
Female morphology PC2 vs genetic distance 1	−0.122	0.845			
Male morphology PC1 vs genetic distance 2	0.070	0.292			
Male morphology PC2 vs genetic distance 2	0.134	0.146			
Female morphology PC1 vs genetic distance 2	0.060	0.326			
Female morphology PC2 vs genetic distance 2	0.114	0.220			
Genetic distance 1 vs genetic distance 2	0.175	0.135			

*P* values indicate the significance of a two-tailed test following 1000 simulations, and boldface type indicates differ significantly (*P* < 0.05). Genetic distance 1 and 2 were inferred from the ND4 gene and SSR, respectively

## Discussion

Lineage separation and divergence form a temporal process in which populations may accumulate genetic, ecological, and/or morphological changes which make organisms better adapt to their environments, until eventually they are reproductively isolated and form separate species [8, 23]. Viviparous *Phrynocephalus* species form a monophyletic lineage that diverged from the oviparous taxa 9.78 Ma, with the most recent common ancestor of viviparous species dated to 5.04 Ma [24]. The species studied herein do not occur in syntopy (Fig. 1). Of these species, only PP has a range overlapping with oviparous congenerics, largely because it evolved earlier than did other viviparous *Phrynocephalus* species currently found on the QTP and at relatively low altitudes allowing oviparous reproduction [22, 24, 25]. The divergence of these species from other viviparous *Phrynocephalus* species on the QTP is dated to 3.79 ± 0.67 Ma, while the earliest speciation event within the complex is dated to 3.09 ± 0.61 Ma [22, 24], following the recent uplift of the QTP (3.6–0.01 Ma). It is therefore likely that environmental changes accompanied by the uplift of the QTP, imposed strong selective forces on local *Phrynocephalus* populations, and promoted morphological and species diversification. In this study, we found weak genetic differentiation but pronounced morphological divergence between species, and that the morphological diagnoses of species boundaries were not supported by genetic evidence. From this study we can draw the following conclusions. First, PG, PP and PV are not reciprocally monophyletic (Fig. 2). Second, morphological divergence is climatically (ecologically) rather than genetically dependent (Fig. 6). Third, PG is genetically and morphologically more similar to PP than to PV (Figs. 2, 3 and 4).

### Weak genetic divergence

Genetic divergence inferred from the ND4 gene was correlated not only with the first climate PC axis (C1) but also with geographic distance (Table 4). This finding allows us to conclude that geographic distance and environmental humidity (or aridity) have major roles in driving genetic divergence between species. In the mtDNA tree, although each species is wildly polyphyletic, we can see similar genetic distance corresponding to the groups identified by morphological characters (Fig. 2). Results of the single Mantel test show a significant correlation between morphological divergence and genetic divergence inferred from the ND4, and significant climatic correlates of morphological and genetic divergence (Table 4). It is worth noting, however, that the morphological-genetic correlation disappeared when holding C1 constant (Table 4). This finding, together with the result that M1 was significantly correlated with C1 in both sexes, indicates that climatic (ecological) dissimilarity rather than genetic divergence has a key role in inducing morphological variation in this group of *Phrynocephalus* species.

Microsatellite-based population genetic analyses showed considerable population level admixture. Twenty-nine out of 462 individuals could be assigned to one of the two identified groups with lower than 70% probability, which supports the occurrence of historical introgressive hybridization at the nuclear genetic level. The unclear assignment between PP individuals from south of Qinghai Lake and PG might result from a lack of geographical barriers, fast and recent population expansion, relatively homogeneous habitat, or a combined effect of these factors.

We found two main monophyletic mtDNA clades that separate populations of PV16, PV20 and PV29 to the rest populations. Noble and his colleagues [20] also found two deeply diverged clades in both mtDNA and nuclear markers that were largely congruent with PV and PP; however, there are many individuals with a nuclear genome composition from one species while with mtDNA haplotype from another in ten sampling sites. The admixed mtDNA clade and the individuals sharing the same haplotypes between species confirm the occurrence of historical introgressive hybridization events between species [20]. Two major genetic clusters in PP were found, respectively corresponding to the Qinghai Lake Basin and the southeast of this basin [19]. PG is genetically very close to PP, as revealed by the fact that the mean pairwise distance between PG and PP was only 2.0% (Figs. 2 and 3). In the mtDNA tree, the lack of resolution of star-shaped clade suggests that these groups diverged quite rapidly. Low genetic diversity and clear pure genetic clustering suggest that PG divergence was a very recent event, presumably as a consequence of adapting to desert environments resulting from the uplift of the plateau. High haplotype diversity and low nucleotide diversity indicate rapid recent population expansion in PG. Additionally, the PCA of climatic variables revealed significant climatic niche separation between species (Fig. 5). This result supports the idea that spatially heterogeneous natural selection can lead to population divergence and ecological speciation even in the presence of gene flow [23].

The admixed mtDNA clade and the individuals sharing the same haplotypes between species imply the occurrence of historical introgressive hybridization events between species. Many hybrids (29/462) with an admixed nuclear genome were detected, and we can expect the presence of individuals with admixed or hybrid genomes as a consequence of hybridization events. In addition, high levels of gene flow between three species suggest that these species may suffer from hybridization. Taken together, the three lines of genetic analyses (mtDNA, STRUCTURE and microsatellite based estimations of migration) all suggest ongoing gene flow between species.

### Adaptive morphological evolution

Species inhabiting different habitats may experience phenotypic divergence in a suite of traits as a result of adaptation to divergent environments [26]. Using different habitats may lead to divergent selection on a number of fitness-related morphological traits, and the morphology-environment correlation has been identified in a number of lizard species [13, 14]. For instance, on the gypsum sand dunes of White Sands, data across three different lizard species show that morphological traits are under strong and multifarious selection, and present evidence of the essential factors for divergence [27].

In this study, morphological differences are evident and show adaptive divergence in response to local environments (habitat type in particular). Similar to the pattern of variation in scale number or size reported for lizards of the genera *Anolis* [28] and *Sceloporus* [14], our data show that species in more arid environments have fewer larger (inferred from the inverse relationship between scale size and number) scales to reduce skin exposure and thus the amount of evaporative water loss (Table 2). Scale number is a heritable trait that is likely to respond to ecologically-based natural selection pressures along environmental gradients, with the complexity of scale hinges, the surface area of skin, and thus the capacity of heat and water exchange increasing with scale number [14, 27, 29]. In agreement with earlier studies of the species studied herein [17, 21], our data show that these species differ morphologically from each other, with body size and scale characters accounting best for morphological divergence between species. Of the three morphological groups, PG and PV are most completely separated, with PP in between (Table 3, Fig. 4). Morphologically, all specimens could be clearly assigned to the species recently described [17, 21]. However, morphological divergence is incongruent with genetic divergence inferred from both mitochondrial and microsatellite DNA data sets, as revealed by the fact that the three species do not form any clear lineages or genetic clusters that can be assigned to individual species already described [17, 21].

Climatic PC scores differed among the three species. Holding geographic distance constant using the partial Mantel test, we found a significant correlation between ecological divergence (climate PC1) and morphological divergence (morphology PC1) (Table 4). These findings suggest that morphological differences between species result from local adaptation. Different habitats can generate strong divergent selection and allow adaptive divergence in space even if gene flow is initially substantial [9, 11–13]. It is therefore likely that these species exhibit morphological divergence due to their differences in habitat preference. Morphological divergence could restrict gene flow, such as by sexual selection linked to morphological traits or coloration [23, 30]. Initial restriction on gene flow could enhance further divergence, and then generate reproductive barriers [31]. Ecological divergence acts as a legitimate isolating mechanism reducing the rate of recombination between divergent habitat types [2], and can therefore drive the evolution of additional intrinsic isolating mechanisms through reinforcement [30].

### Species differentiation process

Environmental changes on the QTP, especially desertification and landcover change, have likely driven population divergence and promoted the speciation of the previously so called *P. vlangalii* (including PP) at 2.29 Ma. After the uplift of the QTP about 1.7 Ma, many areas on the plateau rapidly became arid and some lakes began to disappear [32]. Qinghai Lake became very large during the Middle Pleistocene, caused by the violent lift of the plateau, and then remained stable for a long period due to a drying climate in the Late Pleistocene and declination of its water level since the Holocene [33]. The spreading deserts might have forced *P. guinanensis* to adapt to sand dunes colonized the adjacent area, resulting in the secondary contact of these three species and potential hybridization. Subsequently, recent gene flow may result in convergence at neutral loci, whereas divergent ecology and selection maintain adaptive differences in morphology.

## Conclusions

Our data show that body size and scale characters account best for morphological divergence between species in this group of *Phrynocephalus* lizards. Morphological divergence is related to habitat types that differ climatically. Morphologically, the species boundary is clear between *P. guinanensis* and *P. vlangalii* but not between other two pairs of species. Weak genetic differentiation and pronounced morphological divergence could have resulted from high levels of gene flow and historical introgressive hybridization between species that live in different environments. Our study supports the idea that natural selection in spatially heterogeneous environments can lead to population divergence even in the presence of gene flow. Our study provides a better understanding of genetic, morphological and ecological divergence among closely related species using different habitats, and reveals the initial adaptation to different environments.

## Methods

### Animal collection and treatment

All procedures described in this study were approved by the Animal Care and Use Committee of Nanjing Normal University (2011–04-008). We collected lizards between May and July 2011 from 28 localities around Qinghai Lake (Fig. 1, Table 1). Nine of these localities are occupied only by *P. guinanensis* (PG), 14 by *P. putjatia* (PP), and five by *P. vlangalii* (PV). We identified species based on diagnostic characters reported for these three species [17, 21, 34]. A total of 175 PG (89 females and 86 males), 195 PP (95 females and 100 males) and 106 PV (54 females and 52 males) adults were used for the collection of morphological (11 mensural and 11 meristic characters) data (Table 2). Morphological information for each species × sex × sampling locality combination with a sample size ≥5 was provided in Additional file 1: Table S1. Following the collection of morphological data, the most distal 2–3 mm of the tail tip was excised from each lizard. Lizards were then released at their site of capture. Tissue samples preserved in absolute ethanol were deposited at Nanjing Normal University.

### Mitochondrial DNA amplification and sequencing

Total genomic DNA was extracted from each of 426 individuals using standard phenol-chloroform methods [35]. A partial sequence of the mitochondrial ND4 gene was amplified using forward (ND4) and reverse (Leu) primers [36]. Thermal cycling was performed with initial denaturation for 5 min at 95 °C followed by 35 cycles for 50 s at 95 °C, 45 s at 58 °C, 1 min at 72 °C and a final extension for 10 min at 72 °C. PCR products were purified and sequenced with each of the PCR primers on an ABI 377 sequencer.

### Microsatellite genotyping

We amplified nine microsatellite DNA loci previously developed for *P. vlangalii* (PVMS32, PVMS35, PVMS38 and PVMS39) [37], or for the congeneric *P. przewalskii* (Phr51, Phr75, Phr78, Phr79 and Phr81) [38]. Reactions took place in a thermocycler with an initial denaturation for 5 min at 95 °C followed by 35 cycles for 45 s at 95 °C, 30 s at 57 °C, 40 s at 72 °C, and a final extension for 5 min at 72 °C [39, 40]. Fragment lengths were analyzed with the internal size marker GeneScan-500 ROX (Applied Biosystems), and scored with GeneMarker 2.2.0 (SoftGenetics, LLC, CA, USA).

### Genetic polymorphism

For mitochondrial DNA data we calculated the number of segregating sites, haplotype diversity and nucleotide diversity for each population (locality) and all populations combined using DnaSP 5.10.1 [41]. Fu’s (1997) *Fs* [39] and Tajima’s (1989) *D* [40] were used to detect departures from the mutation-drift equilibrium that could indicate past demographic changes or selection.

For microsatellite DNA data, parameters such as the number of alleles per locus, average allelic richness, observed heterozygosity, expected heterozygosity, the Hardy-Weinberg equilibrium, and exact tests of linkage disequilibrium between pairs of loci for each population were calculated using ARLEQUIN 3.5 [42] and FSTAT 2.9.3.2 [43].

### Phylogeography and population structure

Sequences were aligned using Clustal_X 1.81 [44] with default parameters, and then optimized by eye in MEGA 5 [45]. Mean sequence divergences among major clades were calculated using MEGA 5 and the pairwise Kimura two-parameter (K2P). Bayesian phylogenetic analyses were performed using MrBayes 3.1.2 [46]. We used two oviparous *Phrynocephalus* species as outgroups: *P. albolineatus* (GenBank Accession No. AY054002) and *P. axillaris* (HM235646). Three partitions (the three codon positions of the ND4 sequence; 1st: HKY + G, 2nd: HKY + G and 3rd: GTR + I) were applied to the data and models of molecular evolution were selected for each partition using MrModeltest 2.3 [47]. Four Markov Chains Monte Carlo (MCMC) chains were run for 2.0 × 10^7^ generations. Two independent runs were performed to allow additional confirmation of the convergence of MCMC runs. Two runs from random starting trees resulted in the same topology with negligible differences in clade credibility values. We used NETWORK 4.6.1.0 [48] to generate a median-joining network for all individuals of the three species. To facilitate data presentation and interpretation, we used an initial star-contraction procedure with a star connection limit of two to reduce the data set [49].

We examined each population’s demographic changes by calculating the raggedness index of the observed mismatch distribution according to the population expansion model implemented in ARLEQUIN 3.5 [42]. We used parametric bootstrapping (1000 replicates) in ARLEQUIN 3.5 to test the goodness-of-fit of the observed mismatch distribution. Whether regional or pooled samples matched the spatial expansion model was estimated by the sum of squared deviations statistic.

We used STRUCTURE 2.3.2 [50] to identify genetically distinct groups among microsatellite genotypes with a burn-in of 5 × 10^7^ and 5 × 10^8^ iterations without prior population information, following the admixture model. We conducted 10 replicate runs for each specified value of *K* (the most likely number of populations) from 1 to 20. Individual assignment probability, Ln*P*(*D*) and convergence between runs were used to assess the most likely value of *K*, and the most likely number of clusters was estimated according to Evanno and his colleagues [51].

### Morphological analyses

We used two-way ANOVA [for snout-vent length (SVL) and all meristic variables] or ANCOVA (for other mensural variables with SVL as the covariate) to examine morphological differences between sexes and among species. Prior to parametric analyses, data were tested for the homogeneity of variances using the Bartlett’s test, and for the normality of data using the Kolmogorov-Smirnov test. Tukey’s post hoc test was performed on the traits that differed among species. We performed a principal components analysis (PCA) on 22 morphological variables to show positions of three groups (PG, PP and PV; see Fig. 1 for abbreviation definitions) on a two-dimension plane. For the variables that were related to body size, we removed the size effect by using residuals from the regressions against SVL. All statistical analyses were performed with Statistica 10.0 (Tulsa, OK, USA). Throughout this paper, descriptive statistics are presented as mean ± SE and range, and the significance level is set at *P* < 0.05.

### Ecological divergence

In order to evaluate ecological distinctiveness among groups, we used ArcGIS 10.1 to extract values of the 19 climatic variables available in the WorldClim database (http://worldclim.org/version2) at 30 arc-seconds resolution [52]. In order to remove the effect of colinearity, we performed pairwise correlation comparisons between 19 bioclimatic variables and used nine variables that were not highly correlated (*r* < 0.85) in subsequent analyses. We performed a PCA on the nine climatic variables to reduce the number of predictor variables in our data set, and plotted PC scores on a two-dimension plane. We used linear regression analysis to test if morphology PC scores were correlated with climate PC scores.

### Distance correlation tests

We performed a series of single and partial Mantel tests using the ‘vegan’ package 2.3–5 in R [53] with significance determined using 1000 permutations, testing the correlation between various dissimilarity matrices: (1) geographic distance, (2) climatic PC scores, (3) morphological PC scores, and (4) genetic distance based on mtDNA (genetic distance 1) and microsatellites (genetic distance 2). The climatic and morphological PC scores were converted into distance matrices in R. Only samples that had data for all variables were included in single and partial Mantel tests. Euclidian distance matrices (matrix 2 and 3) were compiled in Statistica 10.0 using the clade means calculated from PC scores for each lizard on a two-dimension plane. Pairwise *F*st values [54] were estimated as the matrix of genetic distance using both mitochondrial and microsatellite data, with the procedure implemented in ARLEQUIN 3.5 [42]. Specifically, we tested the following two hypotheses after accounting for geographic distance between localities: (1) climate predicts morphological differences; and (2) climate predicts genetic divergence.

## Additional files

Additional file 1: **Table S1.** Mensural and meristic data, expressed as mean ± SE and range, collected from adults of three viviparous *Phrynocephalus* species. (XLS 60 kb)

Additional file 2: **Table S2.** Summary of genetic variation at nine microsatellite loci. *H*_o_: observed heterozygosity; *H*_e_: expected heterozygosity; *N*_a_: total number of alleles; *H*_*s*_: genetic diversity; *A*r: allelic richness; *F*_is_: inbreeding coefficients. (DOC 111 kb)

Additional file 3: **Table S3.** Loading of the first two axes of a principal components (PC) analysis on 22 morphological variables for both sexes. Size effects are removed in all cases by using residuals from the regressions on SVL. Variables with the main contribution to each factor are in bold. Species with different superscripts differ significantly (Tukey’s test, α = 0.05; a > b > c). (XLSX 14 kb)

Additional file 4: **Table S4.** Loading of the first two axes of a principal component (PC) analysis on nine climatic variables. (DOC 38 kb)

Additional file 5: **Figure S1.** Median-joining network based on ND4 haplotypes. Red dots in network represent the corresponding mutation steps. Green: *P. guinanensis*; red: *P. putjatia*; blue: *P. vlangalii*. (TIF 5524 kb)

Additional file 6: **Figure S2.** Position of three viviparous species of *Phrynocephalus* lizards in two-dimension space defined by the four principal component analysis according to the mensural and meristic data for both sexes. Green dots and lines: *P. guinanensis*; red dots and lines: *P. putjatia*; blue dots and lines: *P. vlangalii*. (TIF 3711 kb)

## Data Availability

The datasets supporting the conclusions of this article are included within the article and its Additional files 1, 2, 3, 4, 5 and 6. Mitochondrial DNA sequence data have been submitted to GenBank (KJ439240-KJ439557).

## Abbreviations

*A*r: Allelic richness
BSP: Bayesian skyline plot
*F*_is_: Inbreeding coefficients
*H*_e_: Expected heterozygosity
*H*_o_: Observed heterozygosity
*H*_*s*_: Genetic diversity
MCMC: Markov Chains Monte Carlo
*N*_a_: Total number of alleles
PCA: Principal components analysis
PG: *Phrynocephalus guinanensis*
PP: *Phrynocephalus putjatia*
PV: *Phrynocephalus vlangalii*
QTP: Qinghai-Tibetan Plateau
SVL: Snout-vent length
