# Supplementary material for: Genetic and morphological divergence among three closely related Phrynocephalus species (Agamidae)
Source: BMC Evol Biol. 2019 Jun 6;19:114. doi: 10.1186/s12862-019-1443-y (PMC6551896; doi:10.1186/s12862-019-1443-y)
Supplement: Supplementary file 2 — Table S2. Summary of genetic variation at nine microsatellite loci. Ho: observed heterozygosity; He: expected heterozygosity; Na: total number of alleles; Hs: genetic diversity; Ar: allelic richness; Fis: inbreeding coefficients. (DOC 111 kb) [file 12862_2019_1443_MOESM2_ESM.doc]

**Table S2** Summary of genetic variation at nine microsatellite loci. *H*_o_: observed heterozygosity; *H*_e_: expected heterozygosity; *N*_a_: total number of alleles; *H_s_*: genetic diversity; *A*r: allelic richness; *F*_is_: inbreeding coefficients

| Species |  | Phr79 | PVMS35 | Phr75 | PVMS38 | PVMS39 | Phr81 | Phr78 | PVMS32 | Phr51 |
| --- | --- | --- | --- | --- | --- | --- | --- | --- | --- | --- |
| *P. guinanensis* | *H*_o_ | 0.600 | 0.600 | 0.667 | 0.633 | 0.480 | 0.530 | 0.729 | 0.762 | 0.269 |
|  | *H*_e_ | 0.957 | 0.814 | 0.965 | 0.921 | 0.949 | 0.957 | 0.926 | 0.768 | 0.752 |
|  | *N*_a_ | 38 | 30 | 50 | 29 | 35 | 38 | 32 | 26 | 33 |
|  | *H_s_* | 0.958 | 0.814 | 0.966 | 0.922 | 0.951 | 0.958 | 0.927 | 0.768 | 0.753 |
|  | *A*r | 33.477 | 25.288 | 42.644 | 25.344 | 28.592 | 34.048 | 28.104 | 20.870 | 23.602 |
|  | *F*_is_ | 0.374 | 0.263 | 0.310 | 0.313 | 0.495 | 0.447 | 0.214 | 0.008 | 0.643 |
| *P. putjatia* | *H*_o_ | 0.460 | 0.611 | 0.512 | 0.566 | 0.726 | 0.372 | 0.681 | 0.933 | 0.322 |
|  | *H*_e_ | 0.962 | 0.931 | 0.968 | 0.943 | 0.955 | 0.950 | 0.944 | 0.856 | 0.841 |
|  | *N*_a_ | 40 | 33 | 53 | 32 | 37 | 47 | 34 | 25 | 33 |
|  | *H_s_* | 0.963 | 0.932 | 0.969 | 0.944 | 0.955 | 0.951 | 0.944 | 0.856 | 0.842 |
|  | *A*r | 33.223 | 26.398 | 42.229 | 28.568 | 30.740 | 35.819 | 27.467 | 17.868 | 20.096 |
|  | *F*_is_ | 0.523 | 0.344 | 0.472 | 0.401 | 0.240 | 0.609 | 0.279 | -0.091 | 0.617 |
| *P. vlangalii* | *H*_o_ | 0.532 | 0.772 | 0.506 | 0.588 | 0.507 | 0.625 | 0.716 | 0.590 | 0.413 |
|  | *H*_e_ | 0.965 | 0.955 | 0.961 | 0.950 | 0.948 | 0.960 | 0.957 | 0.864 | 0.819 |
|  | *N*_a_ | 41 | 32 | 35 | 30 | 32 | 42 | 30 | 14 | 20 |
|  | *H_s_* | 0.968 | 0.956 | 0.964 | 0.952 | 0.951 | 0.963 | 0.959 | 0.866 | 0.822 |
|  | *A*r | 39.390 | 31.052 | 33.742 | 28.592 | 31.221 | 40.774 | 30.000 | 13.660 | 19.151 |
|  | *F*_is_ | 0.451 | 0.193 | 0.475 | 0.383 | 0.467 | 0.351 | 0.253 | 0.319 | 0.498 |
