# Supplementary material for: Genetic and morphological divergence among three closely related Phrynocephalus species (Agamidae)
Source: BMC Evol Biol. 2019 Jun 6;19:114. doi: 10.1186/s12862-019-1443-y (PMC6551896; doi:10.1186/s12862-019-1443-y)
Supplement: Supplementary file 4 — Table S4. Loading of the first two axes of a principal component (PC) analysis on nine climatic variables. (DOC 38 kb) [file 12862_2019_1443_MOESM4_ESM.doc]

**Table S4** Loading of the first two axes of a principal component (PC) analysis on nine climatic variables

|  | PC 1 | PC 2 |
| --- | --- | --- |
| Annual mean temperature (Bio1) | 0.26 | 0.72 |
| Mean diurnal range (Bio2) | 0.78 | 0.55 |
| Isothermality (Bio3) | **0.93** | 0.01 |
| Temperature Seasonality (Bio4) | −0.27 | **0.92** |
| Temperature annual range (Bio7) | 0.41 | **0.89** |
| Annual precipitation (Bio12) | 0.64 | −0.62 |
| Precipitation of driest month (Bio14) | 0.53 | −0.48 |
| Precipitation Seasonality (Bio15) | **−0.83** | −0.31 |
| Precipitation of driest quarter (Bio17) | **0.84** | −0.41 |
| Variance explained (%) | 43.1 | 37.3 |
