# Supplementary figures and images for: Genetic and morphological divergence among three closely related Phrynocephalus species (Agamidae)
Source: BMC Evol Biol. 2019 Jun 6;19:114. doi: 10.1186/s12862-019-1443-y (PMC6551896; doi:10.1186/s12862-019-1443-y)

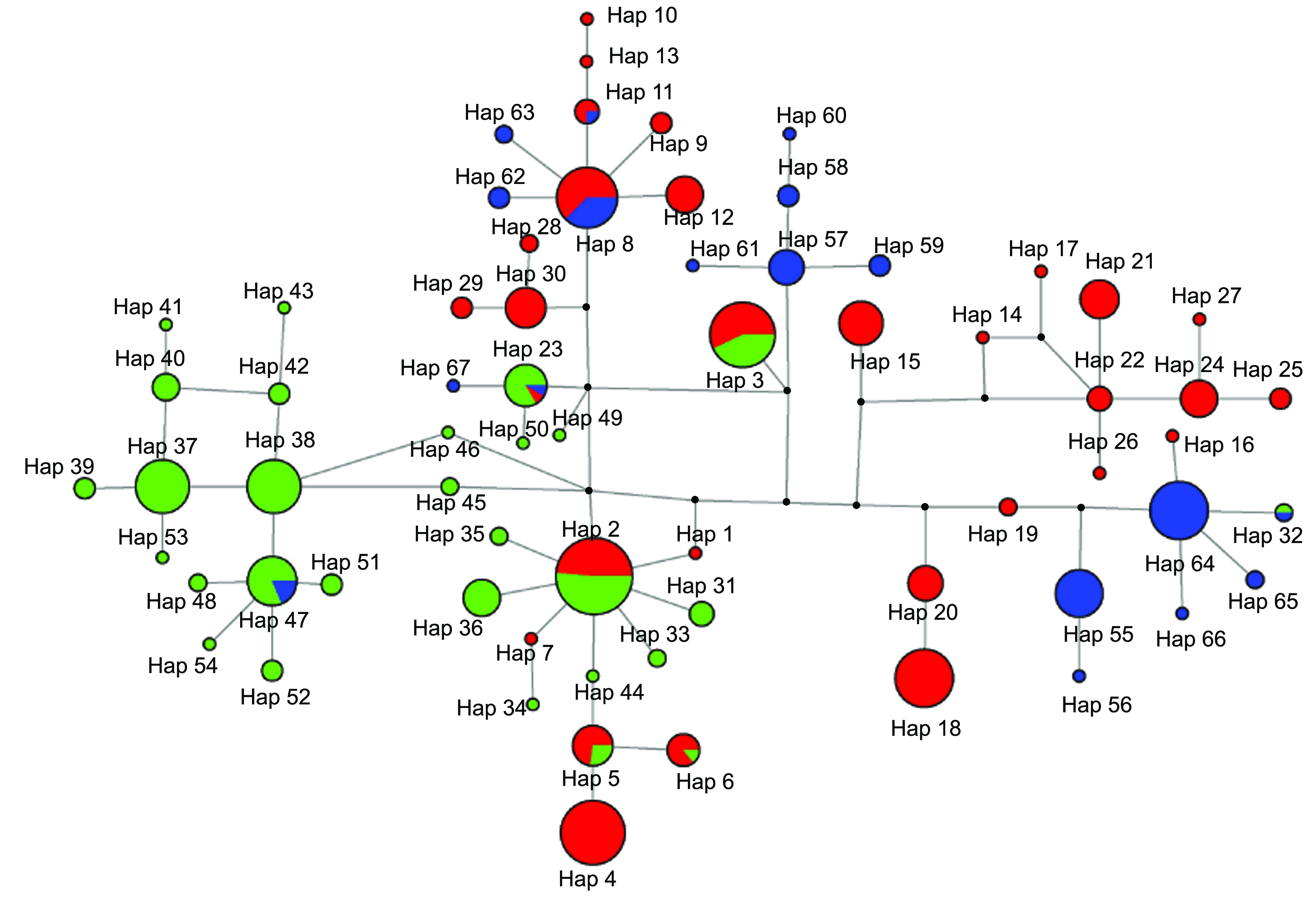

Supplement: Supplementary file 5 — Figure S1. Median-joining network based on ND4 haplotypes. Red dots in network represent the corresponding mutation steps. Green: P. guinanensis; red: P. putjatia; blue: P. vlangalii. (TIF 5524 kb) [file 12862_2019_1443_MOESM5_ESM.tif]

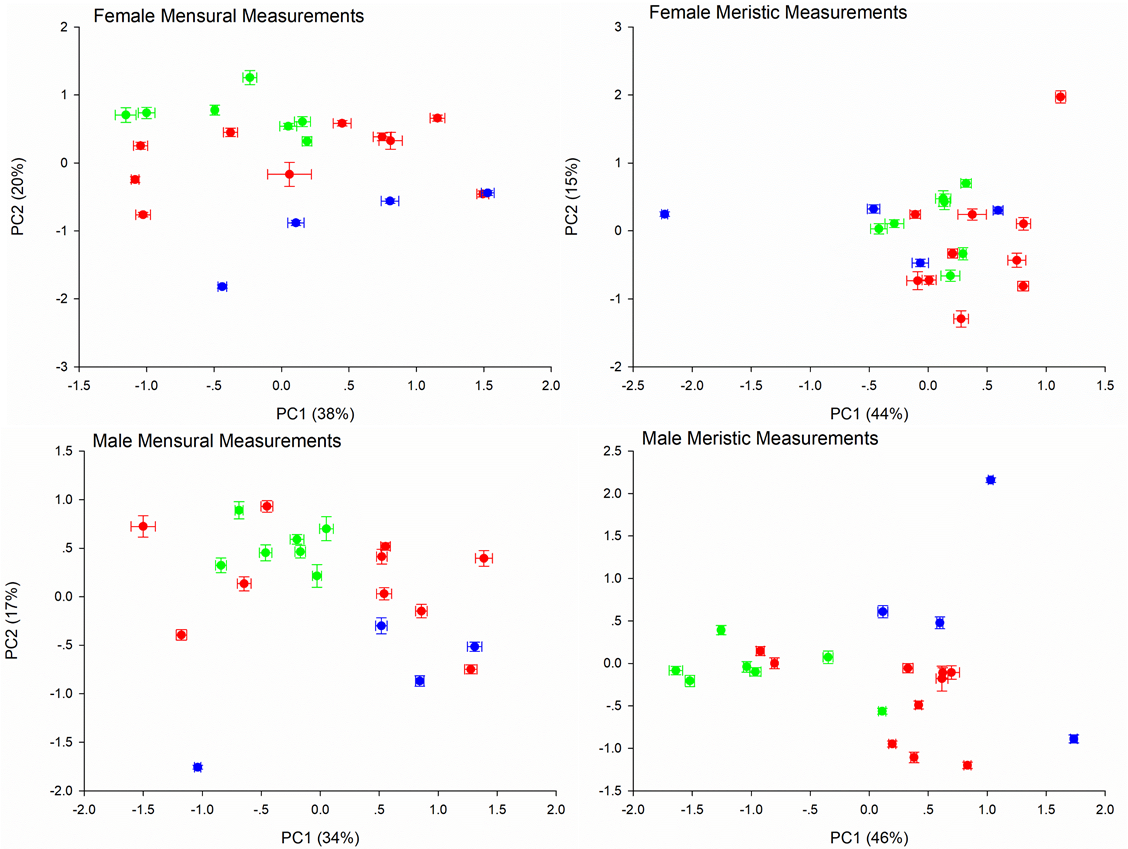

Supplement: Supplementary file 6 — Figure S2. Position of three viviparous species of Phrynocephalus lizards in two-dimension space defined by the four principal component analysis according to the mensural and meristic data for both sexes. Green dots and lines: P. guinanensis; red dots and lines: P. putjatia; blue dots and lines: P. vlangalii. (TIF 3711 kb) [file 12862_2019_1443_MOESM6_ESM.tif]
